# Editorial: Cellular and animal models of neurodegenerative and neuroinflammatory conditions

**DOI:** 10.3389/fncel.2026.1961369

**Published:** 2026-08-31

**Authors:** Juan Antonio García-León, Beatriz García-Díaz, David Baglietto-Vargas, Carlos J. Rodriguez-Ortiz

**Affiliations:** 1 Departamento de Biologia Celular, Genetica y Fisiologia, Facultad de Ciencias, Instituto de Investigacion Biomedica de Malaga-IBIMA-Plataforma Bionand, Universidad de Malaga, Málaga, Spain; 2 Centro de Investigacion Biomedica en Red sobre Enfermedades Neurodegenerativas (CIBERNED), Madrid, Spain; 3 Departamento Fisiología Humana, Histología Humana, Anatomía Patológica y Educación Física y Deportiva, Facultad de Medicina, Universidad de Malaga, Málaga, Spain; 4 Unidad de Gestión Clínica (UGC) Neurociencia, Instituto de Investigacion Biomedica de Malaga (IBIMA)-Plataforma Bionand Málaga, Málaga, Spain; 5 Center for Occupational and Environmental Health, Department of Environmental and Occupational Health, University of California, Irvine, Irvine, CA, United States; 6 Department of Medicine, University of California, Irvine, Irvine, CA, United States

**Keywords:** Alzheimer's disease, animal models, autoimmune encephalitis, cellular models, glial cells, neurodegeneration, neuroinflammation, Parkinson's disease

Glial cells and the immune signaling, they orchestrate have moved from a supporting role to a central position in our understanding of neurodegenerative and neuroinflammatory diseases. Yet translating mechanistic insights from cellular and animal models into effective therapies remains challenging, partially because no single model captures the full complexity of glia-neuron-immune crosstalk across the many aspects involved in health and disease. This Research Topic, “Cellular and Animal Models of Neurodegenerative and Neuroinflammatory Conditions,” was launched to bring together new models that better mimic these processes and to evaluate candidate interventions within them. The five contributions assembled here span enteric and central glia, Alzheimer's and Parkinson's disease, autoimmune encephalitis, and neuroinflammation-associated mood disorders, using *in vitro, in silico*, and *in vivo* approaches, at times within the same study. Together they illustrate both the progress made and the gaps that remain in this fast-moving field.

Within this Research Topic, two contributions extend the modeling of Parkinson's disease (PD) along the gut-brain axis. Samur et al. examined lipopolysaccharide (LPS)-stimulated enteric glial cells (EGCs), a peripheral glial population whose reactivity is increasingly implicated in the “gut-first” hypothesis of PD. When rat-derived EGCs were exposed to the natural diterpenoid oridonin, the compound dose-dependently suppressed LPS-induced upregulation of TLR4 and the activation marker S100B, normalized the transcripts levels of the autophagy-related genes *beclin-1* and *LC3*, and, in combination with the TLR4 antagonist TAK-242, limited IL-1β production more effectively than either compound alone. Molecular docking further suggested that oridonin engages both the extracellular TLR4-MD-2 complex and the intracellular TIR signaling domain, with a stronger predicted affinity than TAK-242 at both sites. The study positions EGCs as a tractable peripheral model for probing gut-derived neuroimmune contributions to early PD, and oridonin as a dual-acting candidate that dampens both inflammatory and autophagic stress.

Complementing this peripheral perspective, Khan et al. modeled the central, protein-aggregation side of PD by combining intra-striatal injection of human α-synuclein preformed fibrils (PFF) with systemic administration of the mitochondrial toxin rotenone in wild type mice. Rotenone acted synergistically with PFF to accelerate the spreading and aggregation of both phosphorylated and proteinase K-resistant α-synuclein in nigral dopaminergic neurons, deepened the loss of these neurons and their striatal terminals, and intensified microglial and astrocytic activation—effects that were more pronounced when rotenone exposure began 3 weeks after fibril seeding rather than 1 day afterward. By coupling an environmental toxicant with a genetically tractable seeding paradigm, this model captures the multifactorial, gene-environment interactions increasingly thought to underline sporadic PD and offers a shorter-latency alternative to purely genetic or purely toxin-based systems.

Moving on to Alzheimer's disease (AD), Camprubí-Ferrer et al. dissected the molecular role of galectin-3 (Gal3) in microglia by generating CRISPR-generated Gal3-knockout BV2 cell line together with exogenous administration of this leptin. Endogenous Gal3 constrained cell size, mitochondrial activity, motility, phagocytosis, and endocytosis, and was required to maintain surface CD11b while restraining the disease-associated microglia (DAM) markers TREM2 and Clec7a. Exogenous Gal3, by contrast, induced CD45 internalization and paracrine TNFα release. To translate these findings into an AD context, Gal3 was deleted in the 5xFAD mice. Deletion resulted in unchanged microgliosis but markedly increased peri-plaque Clec7a+ disease-associated microglia, proposing the Gal3-Clec7a regulatory axis as an important driver contributing to the adoption of detrimental states by microglia in AD. Given that Gal3-targeting antibodies are already undergoing early-phase clinical testing for AD, this granular dissection of which microglial functions are genuinely Gal3-dependent (and which are not) has direct relevance for anticipating the effects of Gal3-directed therapies.

Autoimmune neuroinflammation is addressed by Zeng et al. in a systematic review of active immunization animal models of anti-N-methyl-D-aspartate receptor (NMDAR) encephalitis. The authors classify existing approaches into three families of models: (1) immunization with full-length, conformationally stabilized NMDAR holoreceptors, (2) immunization with GluN1 subunit peptides, and (3) herpes simplex virus (HSV)-triggered models, and weigh their relative capacity to reproduce antigen presentation, T- and B-cell cooperation, blood-brain barrier integrity, and the spontaneous seizures and behavioral phenotypes seen in patients. In this review, the authors conclude that no single model currently reproduces the full clinical picture: the full-length protein approach is technically demanding, peptide models omit T-cell help and rarely generate spontaneous seizures, and HSV-triggered models have so far been validated only by serum antibody titers. The review's comparative framework, and its call for humanized and monoclonal-antibody-based refinements, provides a practical roadmap for selecting or improving a model according to the mechanistic question at hand.

Finally, Bhat et al. described a cellular platform designed explicitly to test a candidate intervention: bioresorbable magnesium-lithium (Mg-Li) alloys proposed as lithium-releasing implants for bipolar disorder and other conditions with a neuroinflammatory component. Using conditioned medium from pro-inflammatory macrophages to drive a microglia-astrocyte coculture toward a reactive state (a model of peripheral inflammation inducing neuroinflammation), the authors showed that lithium delivered via Mg-Li extracts increased the inhibitory GSK3β phosphorylation as effectively as lithium salts, downregulated IL-6 more effectively, and upregulated the neurotrophin *GDNF* more strongly, with excess magnesium not antagonizing lithium's effect on astrogliosis markers. This work exemplifies how a purpose-built neuroinflammation assay can help de-risk a biomaterials-based therapeutic strategy before it reaches an animal model.

Read together, these five studies point to several convergent themes. First, glial reactivity (whether in enteric glia, microglia, or astrocytes) recurs as the shared cellular substrate through which diverse insults (endotoxin, misfolded protein, autoantibody, biomaterial-delivered ion, and environmental toxicant) are translated into neurodegenerative or neuropsychiatric consequences, reinforcing the founding premise of this Research Topic that glia and neuroinflammation are pivotal, cross-cutting players rather than disease-specific curiosities. Second, several of the included studies deliberately combine complementary methodologies: *in vitro* assays with *in silico* docking, genetic cell models with *in vivo* disease models, and environmental with genetic disease drivers. These combinations highlight the need to integrate models and approaches to achieve a robust understanding of neuroinflammation under pathological conditions. Third, the same signaling nodes persist across otherwise unrelated diseases: TLR4 and inflammasome/IL-1β signaling appear in the enteric glia, AD-microglia, and biomaterials studies alike, and receptor-mediated clearance and internalization (Clec7a/TREM2/CD11b/CD45 in AD microglia, NMDAR internalization in autoimmune encephalitis) is a repeated mechanistic thread. Finally, the Research Topic illustrates a broadening therapeutic toolkit under evaluation: small molecules (oridonin), antibody-based strategies (anti-Gal3, NMDAR-blocking antibodies), and biomaterials (Mg-Li alloys); reflecting a field that is diversifying its intervention strategies as fast as it is diversifying its models.

At the same time, the Research Topic makes clear how much remains to be done. Most of the cellular systems described here still rely on immortalized cell lines or short-term primary cultures rather than patient-derived or human pluripotent stem-cell-derived glia, thereby limiting direct extrapolation to human pathology; several authors explicitly identify this as a next step. *In vivo* validation of mechanistic candidates such as oridonin and the Gal3-Clec7a axis, as well as the new PD gene-environment model, will require longitudinal, behaviorally anchored studies rather than the cross-sectional endpoints reported here. The NMDAR encephalitis field, in particular, still lacks a single model that reproduces the full human phenotype, underscoring a broader truth for the field: humanized systems, monoclonal reagents, standardized outcome measures, and imaging- or biomarker-based readouts of disease progression will be needed before preclinical findings of this kind can reliably predict clinical benefit. Integrating peripheral compartments (gut, immune, and biomaterial) with the central nervous system within unified experimental pipelines, as several of these papers begin to do, appears to be a particularly promising direction for future work.

